# Biologics for eosinophilic COPD: current applications and future prospects

**DOI:** 10.3389/fimmu.2026.1722371

**Published:** 2026-02-26

**Authors:** Qi Qi, Wenbei Peng, Bohan Yang, Shijie Liu, Ying Pu, Qiong Zhou

**Affiliations:** Department of Pulmonary and Critical Care Medicine, Union Hospital, Tongji Medical College, Huazhong University of Science and Technology, Wuhan, China

**Keywords:** biologics, COPD, eosinophil, targeted therapy, type 2 inflammation

## Abstract

Chronic obstructive pulmonary disease (COPD) is a heterogeneous pulmonary disorder characterized by persistent airflow limitation and symptoms of progressive dyspnea, cough, and sputum. While traditionally linked to type 1 immunity, a significant subset of patients presents with eosinophil-predominant type 2 inflammation. These individuals remain at risk of exacerbations despite receiving triple therapy, underscoring the need for novel biologics targeting type 2 pathways. Monoclonal antibodies against targets such as IL-5/IL-5Rα, IL-4, IL-13, IL-33/ST-2, and thymic stromal lymphopoietin (TSLP) have shown considerable promise. Therefore, the identification of accurate and accessible biomarkers for type 2 inflammation is crucial. This review summarizes the current applications and future prospects of emerging biologics in eosinophilic COPD, with a specific focus on the role of biomarkers.

## Introduction

Chronic obstructive pulmonary disease (COPD) is a heterogeneous lung disease characterized by persistent respiratory symptoms (dyspnea, cough, sputum production) and/or acute exacerbation due to structural alterations in the airways and/or alveoli, usually accompanied by progressive and largely irreversible airflow limitation (1). COPD represents a major global health challenge, affecting a substantial proportion of the population and accounting for approximately 3.5 million deaths in 2021, which corresponds to nearly 5% of total global mortality (2). Furthermore, it is projected to become the third leading cause of morbidity and mortality worldwide by 2030 (2, 3), thereby imposing a substantial and growing economic burden on healthcare systems across the globe (4). Despite receiving the maximum treatment with triple therapy, approximately half of COPD patients continue to deteriorate (5). Therefore, there is a pressing need for the development of targeted and personalized therapeutic strategies.

Current research in various diseases focuses not only on evaluating the safety of traditional medications (6) but also on developing novel biologics (7). Monoclonal antibodies (mAbs) targeting type 2 inflammation have demonstrated promising therapeutic effects in clinical trials involving COPD patients with high eosinophil (EOS) counts. This review aims to synthesize evidence on targeted therapies for eosinophilic chronic obstructive pulmonary disease (eCOPD), emphasizing biomarker discovery to pave the way for personalized medicine in this field.

## Heterogeneity of COPD and clinical features of the eosinophilic COPD

Chronic inflammation plays a significant role in COPD in both the stable phase and exacerbations. Activation of immune cells such as neutrophils and macrophages, along with pro-inflammatory mediators (8), leads to airway structural changes, airflow obstruction, and respiratory symptoms. The understanding of COPD inflammation has evolved from an initial focus on a type 1 response, mediated by CD4^+^ T helper 1 (Th1) cells and neutrophils, to include the recognition that a significant subset (20–40%) of patients exhibits a distinct type 2 inflammation involving CD4^+^ Th2 cells, type 2 innate lymphoid cells (ILC2s), and eosinophils (9). More recent research has further delineated a type 3 inflammatory endotype, driven by interleukin-17 (IL-17) and IL-22, in certain patients (10).

Although the blood eosinophil count (BEC) in healthy adults exhibits a right-skewed distribution, studies from Japan and China indicate that the median BEC in healthy adults is approximately 100 cells/μL (11, 12). However, patients with high eosinophilia are commonly defined using thresholds of sputum eosinophils ≥3% and/or BEC ≥300 cells/μL (13, 14), exhibiting distinct clinical characteristics. COPD patients with persistently ≥2% eosinophil counts predominantly comprise males, the elderly, former smokers, and those with high body mass index and low BODE index (15). This phenotype is associated with more pronounced upper respiratory symptoms (16), more severe emphysema (17, 18), and an elevated risk of acute exacerbations following the withdrawal of inhaled corticosteroid (ICS) therapy (9). Nevertheless, these patients exhibit accelerated symptom resolution (19) and higher treatment success rates (20), with ICS demonstrating superior efficacy in both the prevention and management of exacerbations within this subgroup (15).

## Challenges and limitations of conventional therapies

Traditional management of COPD integrates both non-pharmacological and pharmacological strategies. Non-pharmacological interventions, such as vaccination, smoking cessation, pulmonary rehabilitation, and non-invasive ventilation, serve an important role in the prevention of disease exacerbations (17). Pharmacologically, long-acting bronchodilators are prioritized over ICS for patients presenting primarily with breathlessness. In cases of acute exacerbations, treatment pathways are guided by BEC. Specifically, triple therapy with a long-acting beta-agonist (LABA), a long-acting muscarinic antagonist (LAMA), and ICS is recommended for patients with a history of exacerbations and a BEC exceeding 300 cells/μL, as it significantly reduces future exacerbation risk (18).

Despite these conventional approaches, approximately half of all COPD patients continue to experience disease progression (5). Several factors contribute to this suboptimal response. For instance, smoking status has been shown to attenuate the beneficial effect of ICS on exacerbation reduction (21). Furthermore, the therapeutic efficacy of ICS is limited by the relative resistance of airway inflammation to corticosteroids (22), restricted anti-inflammatory gene transcription (21, 23), and concerns regarding long-term safety, including elevated risks of non-fatal pneumonia, osteoporosis, and adrenal suppression (24). Consequently, alternative agents such as roflumilast and azithromycin are considered in selected cases.

The clinical course of COPD is further complicated by a high burden of comorbidities, such as cardiovascular disease, diabetes, asthma, osteoporosis, and depression (25). These conditions are driven in part by shared pathophysiological mechanisms, including chronic smoking exposure and systemic inflammation (5). Therefore, novel anti-inflammatory agents that can mitigate COPD progression and its comorbidities are urgently needed.

## Molecular mechanisms of targeted therapy

Type 2 inflammation represents a common pathogenic pathway in several chronic respiratory diseases, such as asthma (26), COPD, allergic rhinitis, and chronic rhinosinusitis with nasal polyps (CRSwNP) (27). The core mechanism is initiated by epithelial barrier disruption, which releases alarmins including IL-33 and TSLP (5). These alarmins activate downstream signaling cascades, notably nuclear factor kappa-B (NF-κB) and mitogen-activated protein kinase (MAPK) pathways (28), which subsequently stimulate both innate immune responses, primarily through ILC2s, and adaptive immunity via Th2 cell activation (26). This coordinated immune activation drives the secretion of the canonical type 2 cytokines: IL-4, IL-5, and IL-13.

IL-4 promotes the differentiation of naïve CD4^+^ T cells into Th2 cells, establishing a positive feedback loop that sustains cytokine production (26). Together, IL-4 and IL-13 induce immunoglobulin E (IgE) class switching in B cells, enhance release of pro-inflammatory mediators, disrupt epithelial integrity, and contribute to tissue remodeling (26). IL-5 exerts a specific and critical role in eosinophil biology by binding to the IL-5 receptor α (IL-5Rα) on eosinophil progenitors in the bone marrow, driving their maturation, survival, and release into the circulation (26). Furthermore, these cytokines collectively upregulate eosinophil-attracting chemokines, such as C-C motif chemokine ligand 26 (CCL26) and thymus- and activation-regulated chemokine (TARC), as well as adhesion molecules including vascular cell adhesion molecule-1 (VCAM-1), thereby facilitating the recruitment and accumulation of eosinophils at inflammatory sites (29).

**Figure 1:**
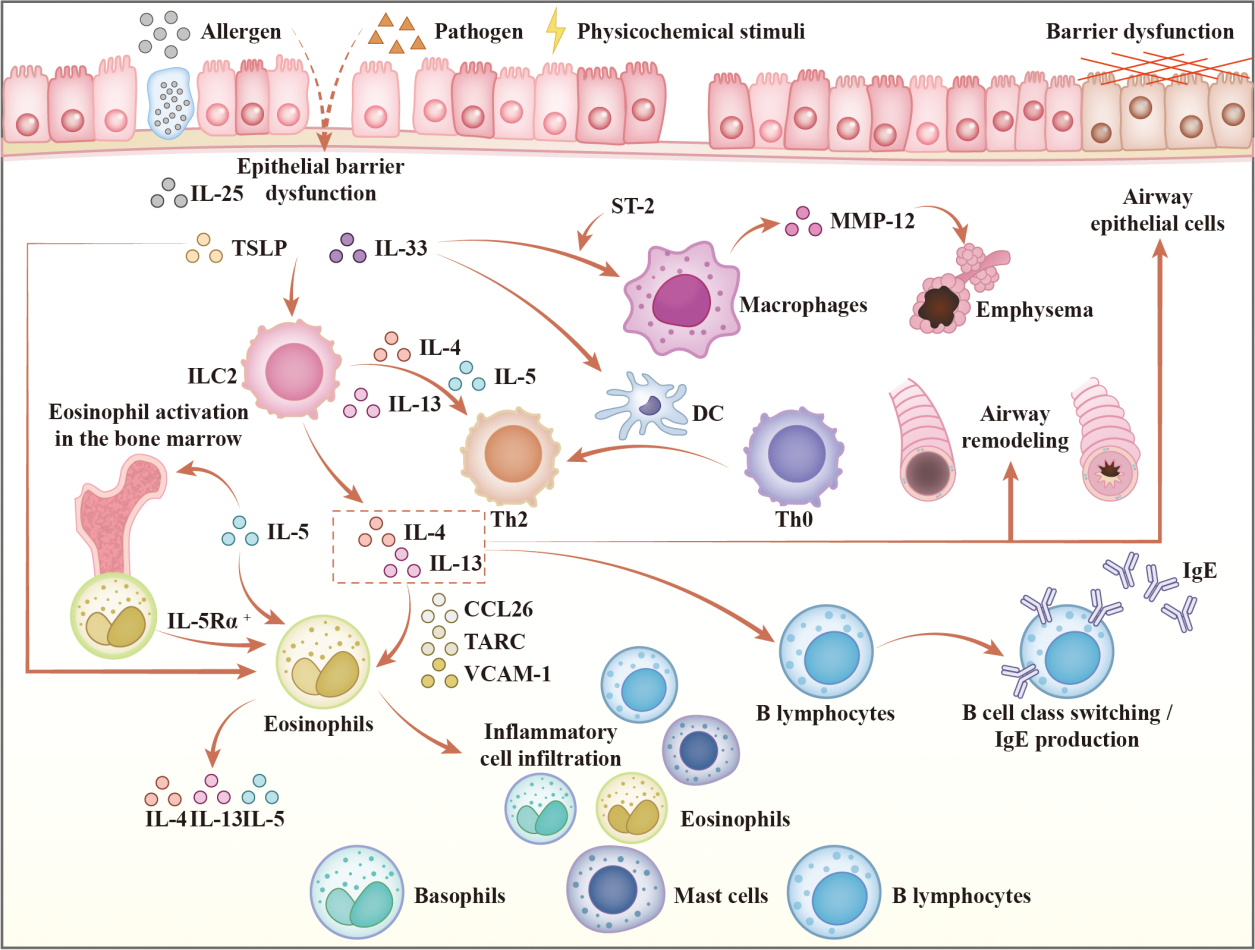
Key mediators and pathways in type 2-high COPD inflammation. TSLP, thymic stromal lymphopoietin; IL-25, interleukin-25; IL-33, interleukin-33; ST-2, suppression of tumorigenicity 2; MMP-12, matrix metalloproteinase-12; ILC2, group 2 innate lymphoid cells; IL-4, interleukin-4; IL-5, interleukin-5; IL-13, interleukin-13; DC, dendritic cells; Th0, naïve CD4+ T cells; Th2, T helper 2 cells; CCL26, C-C motif chemokine ligand 26; TARC, thymus and activation-regulated chemokine; VCAM-1, vascular cell adhesion molecule-1; IgE, immunoglobulin E.

The resultant inflammatory cascade leads to hallmark pathophysiological changes, including airway hyperresponsiveness, epithelial barrier dysfunction, fibrotic remodeling, goblet cell hyperplasia with mucus hypersecretion, and progressive decline in lung function, all of which contribute to chronicity of pulmonary inflammation (5, 30, 31). **Figure 1** provides a comprehensive visual summary of the pathways described above. While biologic therapies targeting type 2 inflammation have demonstrated efficacy in atopic asthma (32, 33), their role in eCOPD warrants focused evaluation. This section elucidates the mechanistic basis of current and emerging targeted agents and discusses their therapeutic potential specifically within the eCOPD phenotype.

## The clinical landscape of targeted therapies

Past attempts to target type 1 inflammation in COPD with biologic therapies have shown limited efficacy (34–38) and have been associated with safety concerns, such as elevated risks of pneumonia and neoplasms (34), which have hindered further clinical development in this area. In contrast, therapeutic strategies focused on type 2 inflammation and its upstream mediators have advanced rapidly. Multiple agents, including monoclonal antibodies against IL-5, IL-5 Rα, IL-4/IL-13, TSLP, and IL-33, have progressed to Phase II clinical trials in COPD (22), with several demonstrating encouraging efficacy and safety signals in early studies (39, 40). The established clinical benefit of analogous biologics in severe asthma supports their continued investigation in eosinophilic COPD and underscores the growing translational potential of type 2-targeted immunomodulation in respiratory disease.

## Biologics in COPD

### Targeting IL-5

IL−5 is a central cytokine governing eosinophilic inflammation (29). Notably, levels of soluble IL−5Rα are elevated during viral−induced acute exacerbations of chronic obstructive pulmonary disease (AECOPD), implicating this axis in exacerbation pathophysiology (41). Blocking the IL−5/IL−5Rα signaling triggers eosinophil death, thereby attenuating eosinophilic inflammation (42), reducing cathepsin−L−mediated extracellular matrix degradation (43), and potentially ameliorating emphysematous changes. Consequently, this mechanistic rationale has guided clinical development, leading to a focus on IL-5/IL-5Rα inhibitors like Mepolizumab and Benralizumab in eosinophil-high AECOPD populations.

Clinical trial outcomes, however, reveal considerable heterogeneity. In selected Phase III trials (e.g., MATINEE [NCT04133909], METREX [NCT02105948]), Mepolizumab demonstrated a significant reduction in the annualized rate of moderate−to−severe exacerbations in patients with BEC ≥300 cells/μL (32, 44). Nevertheless, higher doses did not confer additional benefit, and the METREO study [NCT02105961] failed to show superiority over placebo across its dosing cohorts (44). Similarly, two Phase III trials of Benralizumab (GALATHEA [NCT02138916] and TERRANOVA [NCT02155660]) did not meet their primary endpoint of reducing exacerbation rates in the overall study populations (45). Methodological factors, including inconsistent background medication and the potential inclusion of asthma−COPD overlap patients, may have influenced these outcomes. A post−hoc analysis later suggested that in a predefined subgroup with BEC ≥220 cells/μL, ≥3 exacerbations in the prior year, and receiving triple−therapy baseline, Benralizumab 100 mg may reduce the risk of hospitalization for severe exacerbations (46). This finding was further supported by evidence indicating that Benralizumab significantly lowers severe exacerbation−related hospitalization rates in patients with BEC ≥220 cells/μL (24).

The inconsistent efficacy of IL−5 pathway inhibitors in COPD underscores fundamental limitations of this therapeutic strategy. In stark contrast to their marked effectiveness in severe asthma, these agents demonstrate only selective benefit in COPD, pointing to a more complex, tissue−specific regulation of eosinophilic inflammation in this disease. Critically, even when peripheral eosinophils are effectively depleted (47, 48), key clinical endpoints such as lung function and quality of life often show no significant improvement (32, 44, 49) (**Supplementary Table S1**). This observation directly challenges the simplistic paradigm that eosinophil clearance invariably translates to clinical benefit and aligns with evidence suggesting the existence of an IL−5−independent resident pulmonary eosinophil pool (50). While post−hoc analyses provide hypotheses for refined patient selection, they also highlight the highly conditional real−world applicability of these therapies. Ongoing trials (e.g., SUMMER [NCT05138250], RESOLUTE [NCT04053634]) continue to investigate the efficacy and safety of anti−IL−5/IL−5Rα agents, with the aim of elucidating the precise patient phenotypes that may derive meaningful clinical benefit.

### Targeting IL-4/IL-13

IL-4 and IL-13 play a central role in sustaining type 2 inflammation by promoting the activation and recruitment of type 2 inflammatory cells including eosinophils (5). Furthermore, by suppressing Toll-like receptor-3 expression and signaling, these cytokines impair the antiviral response to rhinovirus-16, leading to increased viral replication (51). This suggests IL-4 and IL-13 contribute to infection-mediated AECOPD.

Lebrikizumab, a monoclonal antibody targeting IL-13, effectively blocks the formation of the IL-13receptor alpha 1 (IL-13Rα1)/IL-4 receptor alpha 1 (IL-4Rα) heterodimeric complex. While it improved lung function and reduced exacerbation rates in asthma trials (52), early data from a randomized controlled trial (RCT) in COPD patients [NCT02546700] found no significant impact on AECOPD incidence (41), leading to the discontinuation of its clinical development for COPD. This efficacy gap underscores intrinsic mechanistic distinctions between type 2 inflammation in asthma and COPD. Despite shared phenotypes such as eosinophilia, the COPD inflammatory milieu is characterized by inherent corticosteroid resistance (53) and a more complex, mixed neutrophilic inflammation, which may attenuate the benefit of selectively targeting IL-13 alone. Given IL-13’s role in airflow limitation and mucus hypersecretion, future evaluation of Lebrikizumab could be reconsidered in symptomatic GOLD Group B patients with only mild exacerbations.

In contrast, Dupilumab, a monoclonal antibody targeting IL-4Rα, inhibits signaling mediated by both IL-4 and IL-13. This dual blockade downregulates inducible nitric oxide synthase expression, reduces Th2 cell differentiation, and attenuates type 2 inflammatory cell recruitment (33). Two Phase III trials in COPD patients with BEC ≥300 cells/μL, BOREAS [NCT03930732] and NOTUS [NCT04456673], demonstrated clear clinical efficacy for Dupilumab (33, 54). It reduced the annualized rate of moderate-to-severe exacerbations by 30% and 34%, respectively, and significantly improved pre-bronchodilator FEV_1_ versus placebo (by 83 mL and 62 mL), with sustained benefits through 52 weeks.

The therapeutic landscape of IL-4/IL-13 inhibition thus presents a striking dichotomy: the failure of Lebrikizumab versus the success of Dupilumab. This contrast highlights that selective IL-13 blockade may be insufficient to overcome the complex inflammatory environment in COPD, whereas dual inhibition of IL-4 and IL-13 signaling may yield synergistic effects. Notably, the magnitude of exacerbation reduction with Dupilumab in COPD appears less pronounced than the 47.7% reduction observed in severe asthma (55). This discrepancy may originate from fundamental differences in the molecular drivers of eosinophilic inflammation between the two diseases. Recent transcriptomic (56) analyses reveal that elevated BEC in asthma correlates with significant expression changes in 1,197 airway epithelial genes, indicative of a robust type 2-high signature. In COPD, however, only 12 genes show association with elevated BEC, with minimal overlap, suggesting that the “eosinophilic phenotype” in COPD may be driven by non-canonical inflammatory pathways. This could partly explain the relatively constrained efficacy of IL-4/IL-13 pathway inhibition in COPD. Furthermore, the demographic composition of these trials, which predominantly enrolled White participants and included a high proportion of males, along with their conduct during the Coronavirus Disease 2019 (COVID-19) pandemic, may have influenced outcomes related to racial variability, study implementation, and exacerbation frequency assessment.

Despite inducing minimal reduction in peripheral blood eosinophil counts, Dupilumab has demonstrated more consistent efficacy in reducing exacerbations and improving lung function than anti-IL-5 monoclonal antibodies, establishing it as the only biologic with high-certainty meta-analytic evidence of benefit in eCOPD (48). Its broad efficacy across multiple type 2 inflammatory conditions, including asthma (55) and atopic dermatitis (57), suggests particular utility for COPD patients with comorbid type 2 diseases. Enhanced treatment response in patients with baseline fractional exhaled nitric oxide (FeNO) >20 ppb (58) further supports its biological rationale, culminating in its recent global approval for uncontrolled COPD with elevated eosinophil counts.

### Targeting IL-33/ST-2

IL-33 binding to its receptor, suppression of tumorigenicity 2 (ST-2), has been implicated in COPD pathogenesis. This interaction stimulates macrophages to produce sputum matrix metalloproteinase-12 (MMP-12), thereby promoting pulmonary emphysema development (59, 60). Furthermore, ST-2 antibodies have been shown to attenuate eosinophilic inflammation, reduce Th2 cytokine levels in bronchoalveolar lavage fluid, and enhance viral clearance (8, 61). Given the role of eosinophilic inflammation and viral infection in exacerbations of type 2 inflammatory COPD, targeting the IL-33/ST-2 pathway presents a highly attractive therapeutic strategy for managing patients with eCOPD.

Itepekimab is a human immunoglobulin G monoclonal antibody targeting IL-33. In murine models, it has been shown to attenuate the infiltration of eosinophils and neutrophils. Notably, Itepekimab not only suppresses established and nascent type 2 inflammation but also prevents its progression to a more severe mixed inflammatory phenotype after 8 weeks. Furthermore, it inhibits the increase in myofibroblasts, thereby mitigating the decline in lung function (62). Currently published data demonstrate variable efficacy profiles for Itepekimab. A phase IIa trial [NCT 03546907] (63) found that while the Itepekimab group showed a modest but significant improvement in pre- bronchodilator FEV_1_(mean increase 0.06L, p=0.024), there was no significant reduction in annualized exacerbation rates (p=0.13). Although baseline BEC was not associated with exacerbation reduction, higher BEC levels predicted a better FEV_1_ treatment outcomes. Subgroup analyses (63) further revealed smoking status significantly influenced efficacy in COPD patients: Itepekimab reduced exacerbation rates by 42% (p=0.0061) and improved lung function (p=0.0076) in former smokers, whereas current smokers showed no benefit in either endpoint. Unlike AERIFY-1 [NCT04701983], where Itepekimab significantly reduced the annualized rate of moderate or severe exacerbations in former smokers, AERIFY-2 [NCT04751487] failed to meet its primary endpoint in a cohort comprising both former and current smokers, showing no statistically significant effect on exacerbation rates (64). This discrepancy in outcomes, observed despite similar baseline blood eosinophil counts, suggests that smoking status may be a critical effect modifier, consistent with the report by Rabe et al. (63).

Tozorakimab, another anti-IL-33 biologic with a dual mechanism of action, was evaluated in a small Phase III trial [NCT04631016] in patients with moderate-to-severe COPD and chronic bronchitis. While it did not significantly improve exacerbation rates or pre-bronchodilator FEV_1_, it demonstrated a significant increase in post-bronchodilator FEV_1_ at 12 weeks (p=0.044), with a more pronounced effect in a subgroup with a history of ≥2 exacerbations (65).

Astegolimab, a fully human anti-ST-2 monoclonal antibody, has yielded mixed results. The Phase IIb ALIENTO trial [NCT05037929] demonstrated a 15.4% reduction in the annualized rate of moderate-to-severe exacerbations, whereas the Phase III ARNASA study [NCT05595642] did not meet its primary endpoint (66). A separate single-center Phase IIa study (COPD-ST2OP, [NCT03615040]) found that Astegolimab only significantly improved health status (mean SGRQ-C difference: -3.3, p=0.039) (67). *Post-hoc* analyses of Astegolimab trials revealed distinct biomarker-response relationships. Specifically, lower baseline eosinophil counts in blood and sputum, combined with elevated serum ST-2 concentrations, correlated with a greater reduction in acute exacerbations. In contrast, more significant improvements in lung function and health status were associated with higher baseline blood eosinophil levels. This divergence in associations, observed even as treatment consistently lowered circulating eosinophil counts, suggests that eosinophils may influence separate pathological processes in COPD. These processes appear to differentially govern exacerbation risk versus airflow limitation and symptom burden, indicating that eosinophils do not constitute a monolithic therapeutic target (68).

The clinical development of IL-33/ST-2 pathway inhibitors in COPD faces significant challenges. Itepekimab shows efficacy limited strictly to former smokers, while Tozorakimab has not yet demonstrated clear clinical advantages despite its dual mechanistic design. Therapeutic targeting of this pathway remains exploratory, and its definitive role in COPD management awaits further validation.

### Targeting TSLP

TSLP is another alarmin derived from epithelial cells that directly acts on eosinophils and ILC2s and influences the antigen presentation of dendritic cells and the differentiation of Th2 cells (69) to promote the production of type 2 inflammatory mediators. Tezepelumab, the first monoclonal antibody targeting TSLP, was evaluated in the phase 2 COURSE trial [NCT04039113] in patients with COPD (70). Although the study did not meet its primary endpoint of significantly reducing exacerbations in the overall population, a prespecified subgroup analysis revealed a statistically significant reduction in the annualized exacerbation rate among patients with baseline blood eosinophil counts (BEC) ≥150 cells/μL (hazard ratio 0.63, 95% CI 0.43–0.93). This treatment effect exhibited a dose–response relationship, with greater benefit observed at higher baseline BEC levels. A subsequent meta-analysis further indicated that Tezepelumab significantly improves pre−bronchodilator FEV_1_ in the eCOPD subgroup, with Singh et al. identifying an inflection point for treatment response around 150 eosinophils/μL (48, 70). Similar to Itepekimab, former smokers receiving Tezepelumab showed a more pronounced, though not statistically significant, reduction in exacerbation frequency compared to current smokers (70).

The statistically significant benefit of Tezepelumab in patients with elevated baseline BEC substantiates TSLP as a promising therapeutic target in this phenotypic subset. These findings posit the need for large−scale prospective trials to definitively establish the clinical role of Tezepelumab in eCOPD and to advance personalized treatment strategies. Beyond monoclonal antibodies targeting single pathways, next-generation biologics are under investigation. Bispecific agents such as HXN-1013 (71) and Lunsekimig (72) represent promising therapeutic strategies with the potential to achieve superior clinical efficacy in patients with eosinophilic COPD.

## Key biomarkers in type 2 inflammation

Airway eosinophilia, as a treatable feature in COPD patients, can be effectively modulated by biologics targeting type 2 cytokines or their receptors. Consequently, the identification of accurate and readily measurable tools for detecting type 2 inflammation is of critical importance (39).

Sputum analysis has not been widely adopted in clinical practice due to operational constraints and potential confounding effects of bacterial infection. Baseline BEC remains the most widely used biomarker for identifying type 2-high COPD, despite being subject to circadian variation, infection, and medication effects (58), and despite ongoing debate regarding its correlation with exacerbation risk (18, 73, 74) and lung function decline (15, 75–77). Nevertheless, multiple studies support its clinical utility: baseline BEC shows moderate correlation with sputum eosinophil percentage (45) and helps predict therapeutic response to ICS/LABA (78), Dupilumab (78), and Mepolizumab (44). Furthermore, a greater increase in BEC during exacerbations relative to the stable state may indicate a higher future exacerbation frequency (79).

Significant heterogeneity exists in the BEC thresholds applied across clinical trials. For example, Dupilumab and Mepolizumab often employ a cutoff of ≥300 cells/μL to enrich for a canonical type 2 phenotype, whereas Benralizumab trials used ≥220 cells/μL to broaden the potential treatable population. The NCT03546907 study applied a threshold of 250 cells/μL for its subgroup analysis. Evidence-based medicine further indicates that optimal thresholds are both drug- and endpoint-specific: ≥150 cells/μL for Mepolizumab (for preventing moderate-to-severe exacerbations) and ≥220 cells/μL for Benralizumab (for reducing severe exacerbations) (24). Consequently, a fixed universal cutoff is clinically inadequate; instead, individualized decisions should integrate the specific biologic agent, treatment goals, and longitudinal BEC trends.

FeNO is widely accepted as a non-invasive and readily reproducible biomarker reflecting airway eosinophilia, complementing BEC. Patients with COPD typically present with mildly elevated FeNO levels (80). Sustained elevation of FeNO is associated with an increased risk of acute exacerbations in stable patients (81) and has been shown to predict improvements in lung function and quality of life following ICS therapy (82). Studies demonstrate that Dupilumab treatment in eCOPD significantly reduces FeNO, with higher baseline FeNO correlating with fewer exacerbations (78). However, FeNO levels are influenced by factors such as smoking (80, 83) and body weight (84), limiting their standalone predictive value. Therefore, FeNO should be integrated with complementary biomarkers such as BEC to optimize diagnostic and prognostic accuracy.

The clinical relevance of serum periostin in type 2-high COPD remains unclear. Although periostin levels are elevated in COPD patients (85) and its concentrations in induced sputum (86) and serum (87) positively correlate with eosinophil counts, studies have not consistently linked it to TSLP expression (87), airway remodeling, or ICS responsiveness (88). While some evidence suggests that high serum periostin may associate with better response to ICS/LABA in stable COPD (86), its value as an independent predictor of exacerbation or hospitalization risk is limited (89), and it is not recommended for routine clinical use.

In COPD, the rs1800925 (-1112C/T) polymorphism in the promoter region of IL-13 has been identified as a risk locus across multiple populations (90), including individuals of Asian, Caucasian (91), and Arab (92) descent. The risk allele contributes to disease progression by modulating IL-13 expression levels (93) and interacting with smoking exposure (90). However, the translation of such genetic markers into clinical practice faces substantial barriers. Genetic testing is not part of routine clinical practice, as it lacks the accessibility, cost-effectiveness, and immediate utility of established biomarkers like blood eosinophil count. Consequently, while genetic studies have advanced our mechanistic understanding of COPD, genetic stratification remains largely investigational and has yet to demonstrate practical value for widespread clinical implementation.

Sputum multi-omics analysis offers a powerful tool for COPD endotyping and precision therapy. The neutrophilic phenotype is characterized by elevated levels of IL-8, IL-1β, and tumor necrosis factor-alpha (TNF-α) (94), whereas the eosinophilic phenotype shows increases in eosinophil cationic protein and periostin (95). Sputum transcriptomics has further identified a molecular signature including genes such as LGALS12, ALOX15, and CLC (96), which outperforms sputum eosinophil count in predicting exacerbations after ICS withdrawal, providing more precise therapeutic guidance. Integrating sputum proteomics, metabolomics, and exhaled volatile organic compound (97, 98) profiling with these approaches holds promise for advancing truly individualized management in COPD. **Table 1** summarizes the key advantages and limitations of each biomarker.

**Table 1:** Biomarkers in type 2 COPD.

Biomarkers	Strengths	Limitations	Predictive value	Clinical interpretation
BEC	Widely accessible and standardized; Validated correlate of sputum eosinophilia (r=0.57); Robust clinical trial evidence for efficacy prediction.	Subject to variability (circadian, steroids, infection); Unclear relationship with long-term lung function decline; Drug-specific thresholds; not interchangeable	High. Key predictive biomarker for treatment response	Drug-specific; Trial-validated thresholds; Monitor longitudinally
FeNO	Non-invasive; Easily reproducible; Reflects airway eosinophilic inflammation.	Subject to variability (smoking status and body weight); Limited predictive data specifically for COPD biologics.	Moderate. Predicts exacerbation risk and ICS response; Potential marker for IL-13 targeted therapy	A supplementary biomarker to BEC; Requires cautious interpretation in smokers.
Serum Periostin	Elevated in COPD patients vs. controls	Conflicting results regarding association with Type 2 inflammation & ICS response in COPD; Limited value in predicting future risk (exacerbation, death).	Low. Not a reliable standalone predictive biomarker in COPD	Currently has limited clinical utility in COPD management.
Genetic Polymorphisms (e.g., IL-13)	Reveals underlying disease mechanisms and susceptibility.	Relationship with eosinophilic COPD endotype not yet validated	To be determined. Associated with COPD risk and lung function decline, but therapeutic predictive value is unclear.	Primarily a research tool at present
Sputum Multi-omics	Provides deep molecular phenotyping; Identifies gene signatures (e.g., GAL3ST2, ALOX15) with high predictive power.	Not routinely available; operational complexity.	Potentially High. May surpass eosinophil count in predicting outcomes (e.g., exacerbations after ICS withdrawal).	A promising tool for future precision medicine; Not yet for routine clinical use.

## Limitations of biological agents

While biologics targeting type 2 inflammation hold promise for COPD, their widespread implementation in clinical practice is constrained by cost−effectiveness, accessibility, and practical barriers.

First, economic accessibility remains a primary challenge. High drug costs and variable reimbursement policies significantly limit patient access. In some healthcare settings, conventional maintenance therapies with lower costs have demonstrated clear reductions in exacerbation frequency and associated healthcare expenditure (99), making them more readily adopted and indirectly diminishing the perceived need for biologics.

Second, precision−medicine frameworks are still evolving. Heterogeneous treatment responses influenced by smoking status, varying BEC thresholds, and the predominance of White participants in existing trials create uncertainty regarding generalizability across diverse populations. To support informed clinical decision−making, it is recommended that trials report both individual−based and event−based numbers needed to treat (NNT) (100), thereby mitigating over−optimistic interpretations and helping patients better appraise the actual meaning of “treatment−related risk reduction” (101). The development of validated quantitative risk−assessment tools is also warranted to guide appropriate biologic use.

Beyond economic and stratification challenges, patient−related and systemic implementation barriers further restrict biologic uptake. Younger patients often prioritize short−term symptom relief (102) and remain cautious toward high−cost, long−term biologic regimens. Adherence is particularly problematic among current smokers, patients with multiple comorbidities, or those skeptical of treatment efficacy (103). Additional practical hurdles (104) include long disease duration, cognitive impairment, the need for repeated injections (105), and physician−related factors such as clinician age and familiarity with biologic therapies.

Notably, smoking status acts as a critical effect modifier for COPD biologics, with its influence being both mechanism−specific and timing−dependent. For example, the IL−33 inhibitor Itepekimab shows significant benefit in former smokers but limited efficacy in current smokers; similarly, the TSLP inhibitor Tezepelumab trends toward greater exacerbation reduction in former smokers. In contrast, Tozorakimab, a dual−mechanism IL−33 inhibitor that blocks both canonical IL−33/ST-2 signaling and prevents oxidative IL−33 inactivation via the RAGE/EGFR axis (106), demonstrated consistent efficacy irrespective of smoking status. The contrasting impact of smoking on the efficacy of Itepekimab and Tozorakimab underscores the specificity of smoke−induced immunomodulation. The limited benefit of Itepekimab in current smokers likely reflects its inability to fully overcome the broad pro−inflammatory effects of cigarette smoke, which may alter IL−33 pathway gene transcription or protein activity (63). In contrast, Tozorakimab achieves its effect through a dual mechanism that simultaneously inhibits IL−33 signaling and promotes epithelial repair, thereby providing broader anti−inflammatory coverage that appears less constrained by smoking status. Preclinical evidence (107) further highlights the importance of timing: anti−IL−1α treatment during active smoke exposure disrupted adaptive responses and led to rebound inflammation upon cessation, whereas the same treatment initiated after smoke withdrawal accelerated inflammation resolution without rebound. This observation implies that the therapeutic window for biologic intervention may be shared across different inflammatory pathways and strongly modulated by smoking status.

Moving forward, systematic evaluation of smoking as an effect modifier is essential. Future trials should pre−specify stratification by smoking status and explore the pharmacodynamic differences of targeted therapies in specific smoking subgroups. Such efforts will be crucial for refining patient selection and ultimately achieving precision application of biologics in COPD.

## Conclusion

In summary, type 2 inflammation plays a pivotal role in the pathogenesis of eosinophilic chronic obstructive pulmonary disease. A growing number of biologics targeting type 2 inflammatory pathways have garnered significant interest and undergone extensive investigation. Monoclonal antibodies against IL-5, IL-4, IL-13, IL-33, TSLP, and related pathways have demonstrated the potential to reduce acute exacerbation rates, improve lung function, and enhance quality of life, representing a move toward precision and individualized therapy in COPD.

Nevertheless, biological agents encounter several challenges in clinical practice. First, therapeutic efficacy remains inconsistent. Factors such as smoking status, history of exacerbations, and the threshold definition for high eosinophil counts significantly influence treatment response. Second, accurate and readily measurable biomarkers are still lacking, making it difficult to identify COPD subtypes early and administer targeted therapy precisely. Third, long-term safety requires further evaluation (6). Although no significant differences in severe adverse event risks exist between biologics, there is no evidence of sustained efficacy after discontinuation in asthma. Additionally, potential paradoxical responses and infections in COPD patients following long-term use warrant vigilance. Fourth, as emerging therapeutic agents, biologics face economic constraints. Limited disposable income and the costs associated with prolonged treatment regimens and close follow-up limit the widespread adoption of monoclonal antibodies. Fifth, the timing and target population for biologic therapy remain inadequately defined. Current clinical studies predominantly involve white populations and employ triple or dual combination therapies as background treatments. The appropriate timing for monotherapy with biologics requires substantial prospective research. Looking ahead, the development of novel agents with validated clinical efficacy, coupled with health-economic evaluations to support broader accessibility, is expected to provide new therapeutic pillars for COPD management.
