# Supplementary material for: Biologics for eosinophilic COPD: current applications and future prospects
Source: Front Immunol. 2026 Feb 26;17:1722371. doi: 10.3389/fimmu.2026.1722371 (PMC12979430; doi:10.3389/fimmu.2026.1722371)
Supplement: Supplementary file 1 [file Table1.docx]

**Supplemental Table 1.** Comparative Analysis of Biologics for Eosinophilic COPD.

| Target | Inhibitor | Study | Blood Eosinophils | Asthma | Patient characteristics | Annual exacerbation rate | Time to the First Moderate or Severe Exacerbation | Symptom assessment | Lung function | Safety | Others |
| --- | --- | --- | --- | --- | --- | --- | --- | --- | --- | --- | --- |
| IL-5 | Mepolizumab  100mg q4w | MATINEE  NCT04133909 | ≥300 cells/μL at visit 0 and a documented historical BEC of ≥ 150 cells/μL | Excluded | current or former smokers, 804 patients | Rate reduction:  0.21 events/year  (RR =0.79, P=0.01) | 419 days vs 321 days, (HR=0.77, P=0.009) | CAT and SGRQ were NSD | NSD | NSD |  |
|  | Mepolizumab  100mg q4w | METREX  NCT02105948 | ≥150 cells/μL at visit 0 or a documented historical BEC of ≥ 300 cells/μL | Excluded | current or former smokers, 462 patients | Rate reduction:  0.31 events/year  (RR =0.82, P=0.04) | 192 days vs 141 days (HR=0.75, P=0.04) | CAT and SGRQ were NSD | No change compared to the baseline | NSD |  |
|  |  |  | all patients, with no eosinophil criteria applied | Excluded | current or former smokers, 836 patients | NSD | NSD | CAT and SGRQ were NSD | No change compared to the baseline | NSD |  |
|  | Mepolizumab  100mg q4w or 300mg q4w | METREO  NCT02105961 | ≥150 cells/μL at visit 0 and a documented historical BEC of ≥ 300 cells/μL | Excluded | current or former smokers, 674 patients | NSD | NSD | CAT and SGRQ were NSD | No change compared to the baseline | NSD |  |
|  | Mepolizumab  100mg q4w | COPD-HELP NCT04075331 | At least one BEC ≥ 300 cells/μL in the 12 months prior to enrolment | Excluded, 5.0% with asthmatic features enrolled | current or former smokers, 238 patients | - | NSD | CAT and SGRQ were NSD | - | NSD | Eosinophils reduced; Time to hospitalization/death: NSD |
|  | Benralizumab  30mg or 100mg q8w | GALATHEA  NCT02138916 | ≥220 cells/μL at visit 0 | Included (low proportion) | current or former smokers, 1120 patients | NSD | - | SGRQ was NSD in 30 mg; SGRQ was SD in 100 mg | No change compared to the baseline | NSD | Eosinophils reduced; Efficacy independent of baseline BEC |

| Target | Inhibitor | Study | Blood Eosinophils | Asthma | Patient characteristics | Annual exacerbation rate | Time to the First Moderate or Severe Exacerbation | Symptom assessment | Lung function | Safety | Others |
| --- | --- | --- | --- | --- | --- | --- | --- | --- | --- | --- | --- |
|  | Benralizumab  10mg , 30mg or 100mg q8w | TERRANOVA  NCT02155660 | ≥220 cells/μL at visit 0 | Included (low proportion) | current or former smokers, 1545 patients | NSD | - | SGRQ was NSD | No change compared to the baseline | NSD | Eosinophils reduced; Efficacy independent of baseline BEC |
| IL-4Rα | Dupilumab  300mg q2w | BOREAS NCT03930732 | ≥300 cells/μL at visit 0 | Excluded | current or former smokers, 939 patients | Rate reduction:  0.32 events/year  (RR =0.70, P<0.001) | - | SGRQ was SD (P=0.002); E-RS–COPD was SD (P=0.001) | Pre-BD FEV_1_ increase vs control: 83 mL(P<0.001) at week 52 | NSD | Greater efficacy with baseline FeNO ≥20 ppb |
|  | Dupilumab  300mg q2w | NOTUS  NCT04456673 | a documented historical BEC of ≥ 300 cells/μL | Excluded | current or former smokers, 935 patients | Rate reduction:  0.44 events/year  (RR =0.66, P<0.001) | 29% risk reduction (HR 0.71, 95% CI 0.57-0.89) at week 52 | SGRQ was NSD | Pre-BD FEV1 increase vs control: 62 mL(P=0.02) at week 52 | NSD | HR 0.51 for first severe exacerbation |
| IL-33 | Itepekimab  300mg (150mg q2w) | NCT03546907 | Subgroup analysis by baseline eosinophils (≥250 vs. <250 cells/μL) | Excluded | current or former smokers, 343 patients | NSD. Rate reduction: 42% only in former smokers (P=0.0061) | NSD | - | Pre-BD FEV1 increase vs control: 60 mL(P=0.024) at week 16-24, benefit driven by former smokers. | NSD | BEC not predictive of exacerbations; FEV₁ benefit in high BEC (≥250 cells/μL); former smokers: fewer severe exacerbations. |
|  | Itepekimab  q2w or q4w | AERIFY-1  NCT04701983 | - | Excluded | former smokers, 1127 patients | Rate reduction: SD (RR=0.73) | - | - | - | NSD |  |
|  | Itepekimab  q2w or q4w | AERIFY-2  NCT04751487 | - | Excluded | current or former smokers, 953 patients | NSD | - | - | - | NSD |  |
|  | Tozorakimab  600mg q4w | FRONTIER-4  NCT04631016 | Subgroup analysis by baseline eosinophils (≥150 vs. <150 cells/μL) | Excluded | current or former smokers, 135 patients | NSD | - | SGRQ was NSD | Post-BD: SD; Pre-BD: NSD | NSD | Eosinophils reduced; Consistent across smoking status; FEV₁ improved in high-risk/BEC≥150 cells/μL |
| ST-2 | Astegolimab q2w or q4w | ALIENTO  NCT05037929 | - | Excluded | current or former smokers, 1301 patients | Rate reduction: SD (RR=0.74) | - | - | - | NSD |  |

| Target | Inhibitor | Study | Blood Eosinophils | Asthma | Patient characteristics | Annual exacerbation rate | Time to the First Moderate or Severe Exacerbation | Symptom assessment | Lung function | Safety | Others |
| --- | --- | --- | --- | --- | --- | --- | --- | --- | --- | --- | --- |
|  | Astegolimab  q2w or q4w | ARNASA NCT05595642 | - | Excluded | current or former smokers, 1375 patients | NSD | - | - | - | NSD |  |
|  | Astegolimab  490mg q4w | COPD-ST2OP  NCT03615040 | - | Included | current or former smokers, 81 patients | NSD | - | SGRQ was SD (P=0.039) | NSD | NSD | No difference by smoking status or BEC |
| TSLP | Tezepelumab  420mg q4w | COURSE NCT04039113 | Subgroup analysis by baseline eosinophils (>300 vs 150-300 vs. <150 cells/μL) | Excluded | current or former smokers, 333 patients | NSD. Rate reduction: SD (RR 0.63) in BEC ≥150 cells/μL | NSD | CAT and SGRQ were NSD | Pre-BD FEV1 increase vs control: 55 mL(P=0.024) at week 52 | NSD | Treatment effect on exacerbations consistent across smoking status subgroups |

Abbreviations: IL, interleukin; BEC, Blood Eosinophil Count; Excluded, Excluded patients with current diagnosis or history of asthma; RR, Rate Ratio; HR, Hazard Ratio; CAT, COPD Assessment Test; SGRQ, St. George's Respiratory Questionnaire; NSD, No Significant Difference; Included, Included patients with current diagnosis or history of asthma; SD, Significant Difference; E-RS-COPD, Evaluating Respiratory Symptoms in COPD; Pre-BD, Pre-Bronchodilator; FEV1, Forced Expiratory Volume in 1 second; FeNO, Fractional exhaled Nitric Oxide; Post-BD, Post-Bronchodilator.
